# Linking product design and durability: A review and research agenda

**DOI:** 10.1016/j.heliyon.2022.e10734

**Published:** 2022-09-22

**Authors:** Jaime A. Mesa, Arturo Gonzalez-Quiroga, Marina Fernandes Aguiar, Daniel Jugend

**Affiliations:** a Department of Mechanical Engineering, Universidad del Norte, Barranquilla, Colombia, 081001; b Department of Industrial Engineering, Pontificia Universidad Javeriana, Bogotá DC, Colombia 110231; c UREMA Research Unit, Department of Mechanical Engineering, Universidad del Norte, Barranquilla, Colombia, 081001; d São Paulo State University (UNESP), Production Engineering Department, Bauru, Brazil, 17000-000

**Keywords:** Product design, Durability, Circular economy, Sustainable design, Product lifecycle

## Abstract

Durability has become a valuable design aspect for designers, manufacturers, service providers, and end-of-life actors. Nowadays, developing products for new business models based on renting and servitization practices is of paramount importance. Furthermore, durability enables the application of circularity strategies for product lifespan extension, including reuse, repair, refurbish, and remanufacture. However, despite the growing trend around durability, there is no precise tracing of its evolution, implementation, and potential benefits from the product design stage. Therefore, this article aims to analyze the existing literature about durability and its relationship with the circular economy concept starting from the product design process, uncovering potential research directions, challenges, and trends for its application. A total of 147 articles were selected and analyzed from 40 years of research using two main approaches. First, a keyword-based analysis was used to identify trending topics around the concept of durability. Second, a content-based analysis was used, encompassing four main aspects: objectives and methodology; actors involved and lifecycle phases; circular economy strategies; and design phase, design attributes, and type of products involved. The analysis identified how the concept has evolved during the last four decades, indicating that future trends envisage methodologies, assessment tools, and guidelines to support product life extension.

## Highlights

• Product durability has evolved from mechanical performance analysis to sustainability issues.
• The durability of components and products is central to circular economy strategies.
• Design methods and metrics are required to design and assess durable products.
• Industry 4.0 is vital to promote and boost product-service systems.

## 1 Introduction

Product durability and, consequently, lifetime extension have been gaining importance during the last two decades, particularly in the evolving fields of design for sustainability (Fiksel, 2009), eco-design (Bundgaard et al., 2017), and the circular economy (CE) *e.g* (Bocken et al., 2016; Ertz et al., 2019; Franco, 2019). In contrast to the current linear production and throw-away patterns, new design concerns focus on closing material loops to preserve products, parts, and materials in the industrial system at their highest utility and value (Zink and Geyer, 2017). Product durability has been one of the main themes in the field of product development for CE since it allows the adoption of strategies such as reuse, repair, refurbish, and remanufacture for product lifetime extension with less environmental burden than recycling (Cramer, 2014; Kirchherr et al., 2017).

Product lifetime extension is also closely related to product durability, which refers to “… *the ability of a product to perform its function at the anticipated performance level over a given period (number of cycles - uses - hours in use), under the expected conditions of use and under foreseeable actions*” (Boulos et al., 2015). As pointed out by den Hollander et al. (2017, p. 521) (den Hollander et al., 2017), “*durability is a physical property of a product,*” and it is highly dependent on the material (Sauerwein et al., 2019). Furthermore, Cordella et al. (2021) (Cordella et al., 2021)indicated two factors that influence the durability of a product: first, the reliability, which is related to how likely the product is to function as specified without any issues occurring, and second the repair processes, *i.e.* returning the product to a functional state.

A product is durable when its degradation takes longer than similar, comparable products (den Hollander et al., 2017). It is possible to achieve this process of slowing down a worsening performance over time by incorporating attributes to avoid damage and promoting the role of the users in maintaining the value of these products (Singh et al., 2019). However, there is a lack of understanding of the relationship between product durability and design for sustainability, especially within the CE framework.

The development of circular flows of materials for products is being encouraged to reduce waste and conserve resources. As Vermunt et al., (2019) (Vermunt et al., 2019) pointed out, one of the many benefits that firms can obtain from the CE implementation is increased independence from external resources. Moreover, CE can also result in economic profits, potentially increasing the Gross Domestic Product (GDP). These possible economic gains are even more relevant in emerging economies, which are more likely to use materials intensively (Ellen MacArthur Foundation, 2015). In this context, the design of more durable products can extend product lifetimes, decreasing resource consumption, saving materials, and reducing waste production (Stamminger et al., 2020). Legislation initiatives, especially in the European Union (EU), e.g., The EU Action Plan for the Circular Economy 2020 (European Comission, 2020) and the Ecodesign Directive 2009 (European Parliament, 2009), have actively contributed during the last decade to the field of sustainability and durable products (Alev et al., 2019). Governments have started to adopt regulations and laws regarding the design and manufacturing of durable goods, attempting to reduce waste and promote products' extended life (Gümüş et al., 2013; W. Zhou et al., 2017). Hence, considering product durability will become essential for governments, companies, and individuals striving for sustainable development.

A thorough literature search yielded six review articles related to product durability. Fontana et al. (2021) (Fontana et al., 2021) analyzed CE strategies that extend machinery lifetime. This research included a strategy characterization framework to select the most suitable strategy according to the situation. Khan et al. (2018) (Khan et al., 2018) explored existing literature regarding product upgradability as a critical strategy for extending product lifetime. This review discussed the potential for upgradeability in product-service systems (PSS) and highlighted the need for specific design approaches for PSS.

Additionally, Zallio and Berry (2017) (Zallio and Berry, 2017)reviewed design theories and practices regarding how to face the issue of built-in obsolescence. This study underlined design as a critical stage to promote the extended life of products. Rivera and Lallmahomed (2016) (Rivera and Lallmahomed, 2016) reviewed premature obsolescence and its implications on the environmental impact of products, calling for attention to the broader impacts of product lifetime throughout the product lifecycle and their incorporation into business models. Maitre-Ekern and Dalhammar (2016) (Maitre-Ekern & Dalhammar, 2016) examined the political efforts to promote durability and reparability in Europe. This study analyzed policies, legal initiatives, and rules to reduce final disposal environmental impacts. Finally, Ertz et al. (2019) (Ertz et al., 2019) comprehensively analyzed product lifetime and business models. This investigation provided a taxonomy of business models for product lifetime extension and a state-of-the-art summary of how companies and consumers extend product usefulness over time.

The missing link between product design and CE is most noteworthy from these reviews. Developing products with high physical and emotional durability can contribute to avoiding obsolescence. Considering durability from the product design stage holds the potential to prolong the use cycle and consequently extend the product lifetime (Aguiar et al., 2022; den Hollander et al., 2017). In this sense, incorporating durability into product design requires shifting from the current product-centric focus toward a more system-based design approach. Extending product lifetime is essential for reusing, repairing, refurbishing, and remanufacturing, which leads to enhanced environmental sustainability and increased added value. Here, durability is crucial in supporting product longevity through durable geometries and materials. While the literature has shed light on design for durability, it has not investigated emotional durability, which encompasses the user experience (den Hollander et al., 2017; Haines-Gadd et al., 2018).

Another aspect investigated by recent literature is the role of digital technologies in supporting the transition towards CE, including improving product design (Bressanelli et al., 2018). For example, from a case study in 10.13039/501100015698LED lighting, Ingemarsdotter et al. (2020) (Ingemarsdotter et al., 2020)concluded that IoT could support design for durability. First, it can provide data related to the use of the products, offering opportunities to rethink the product design and minimize faults. Additionally, IoT enables estimating how long the product has advanced through its lifetime.

Besides contributing to the contemporary debate on design for product durability from a CE perspective, this article presents the current scopes in design for product durability, pointing out research gaps and future research directions. Prior literature reviews have focused on the environmental impact of products from the perspective of obsolescence and business models. Consequently, several research gaps still hinder a proper understanding of product durability and its role in CE:

• None of them have considered implications from the product design perspective, which significantly influences environmental impacts during the product lifecycle (Laurenti et al., 2015; Waage, 2007).
• Designing CE products entails analyzing material and product durability to promote lifetime extension strategies. There is a research gap between CE and product durability, considering both benefits and limitations.
• It is also necessary to analyze the drivers and barriers of product durability approaches to establish future research opportunities. Product durability is related to functional performance, emotions, and consumer behavior (Haines-Gadd et al., 2018).

Through a systematic review, this study intends to address these research gaps regarding product durability and its role in CE. The aim is to systematically review the body of knowledge regarding product durability, seeking to provide new insights and suggest a research agenda.

## 2 Method

### 2.1 Systematic literature review design

First, an exploratory search identified relevant keywords and research fields to narrow the analysis. We then conducted a systematic literature review using the Preferred Reporting Items for Systematic Reviews and Meta-Analyses (PRISMA) guidelines (Moher et al., 2009). PRISMA is a well-established methodology that offers a consistent and effective step-by-step process to help synthesize state-of-the-art literature reviews (Liberati et al., 2009). Besides providing a robust structure to identify, select, and analyze published studies, the main advantage of using PRISMA is that it helps avoid bias in scrutinizing the review. PRISMA provides transparency for literature reviews, has a formal step-by-step guide to identify and analyze previous literature from general to specific scopes, permits the database search process to be reproduced, and avoids typical bias in literature reviews.

**Figure 1:**
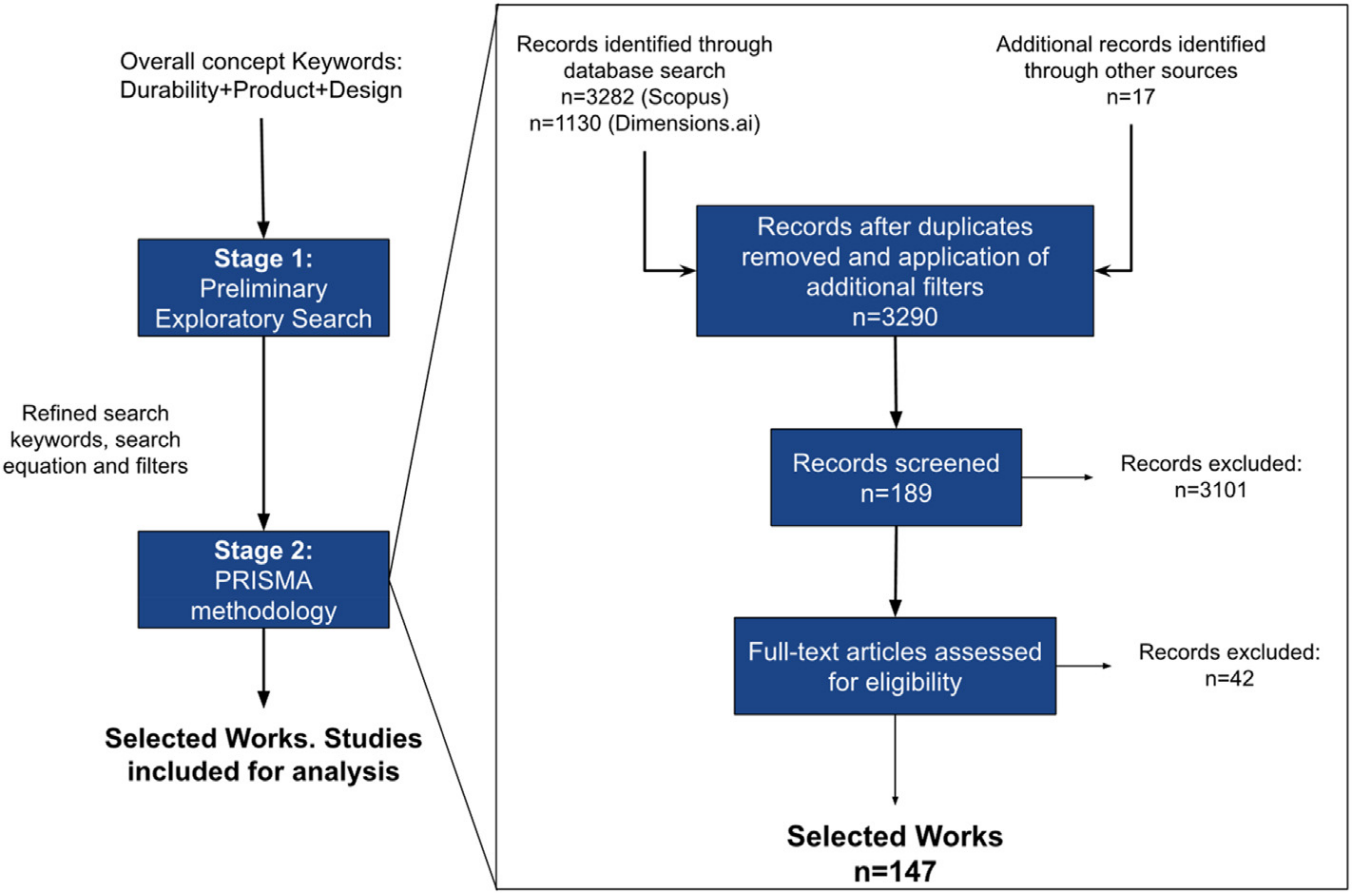
The review process following the PRISMA methodology.

Published literature reviews in the field of CE, such as Alhawari et al. (2021) (Alhawari et al., 2021)and Romero-Luis et al. (2021) (Romero-Luis et al., 2021), have already successfully applied PRISMA. Figure 1 depicts the methodology for the literature search and screening.

#### 2.1.1 Identification

The exploratory search used “product” and “durability” as keywords. However, results showed some unrelated topics because the term “durability” appears in different contexts *(e.g.* in civil construction, regarding the durability of concrete; biology and medicine, related to bacteria life; illnesses and aging; among others). Therefore, a round of preliminary database searches was performed to identify unrelated terms to generate a refined query focused on product durability in product design. Such searches were based on the presence of unrelated words due to limitations for analyzing phrases and sentences and after detecting the research topics commonly related to biology and medicine disciplines.

Subsequently, to apply the PRISMA methodology, we employed a set of refined keywords in the Scopus database, which provided 3,282 entries: “product; ” “design; ” “extended; ” “lifetime; ” “lifespan; ” “durability; ” “obsolescence; ” and “longevity.” We chose the Scopus database because of its rigorous indexing and its extensive collection of scientific journals (Mongeon and Paul-Hus, 2016). A complimentary search in the Dimensions. ai database using “product” and “durability” yielded 1,130 additional entries. The search query is presented in Appendix A.

#### 2.1.2 Screening

Excluding duplicates reduced the number of articles to 3,290. Then we applied two additional filters: journals and conference articles (original articles, reviews, and proceedings); and the English language. An assessment of title, keywords, and abstract enabled the screening of the resultant 3,101 articles, following which 189 articles reached the “eligibility and inclusion” stage.

#### 2.1.3 Eligibility and inclusion

At this stage of the PRISMA method, the eligibility criterion was peer-reviewed works that include, measure, assess, or determine product durability during any stage of the product lifecycle. The final set comprised 147 eligible papers. We highlight that grey literature and web-based reports, such as those of the Ellen MacArthur Foundation (EMF) and the work of consultancies such as McKinsey, were not used in this review, considering that, as pointed out by (Lindgreen et al., 2020), the origin of these documents falls beyond the academic community. Furthermore, to our knowledge, no database gathers this type of grey literature. Therefore, to maintain a systemic search approach, following the scope of this research, these documents were not used.

The readers interested in a deeper understanding of CE related to extended product lifespan from gray literature can consult the Ellen MacArthur Foundation (https://archive.ellenmacarthurfoundation.org/explore/circular-design), WBCSD (https://www.ceguide.org/Strategies-and-examples/Design/Lifetime-extension-durability), or ONE PLANET 2021 (https://www.oneplanetnetwork.org/programmes/consumer-information-scp/product-lifetime-extension).

#### 2.1.4 Descriptive characteristics of the sample

**Figure 2:**
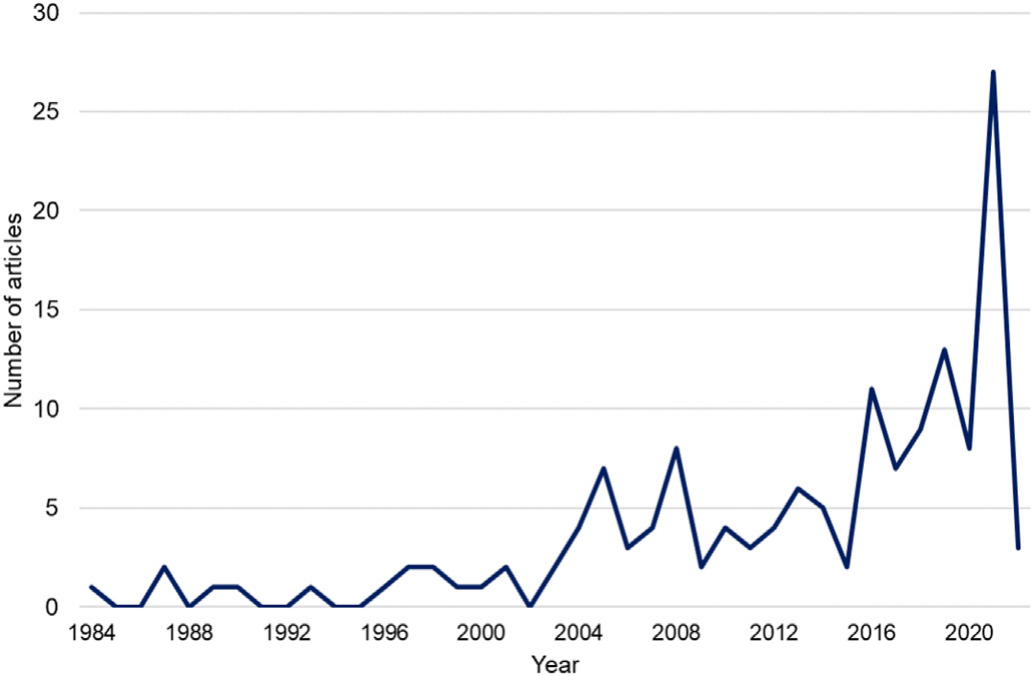
Time evolution of research papers related to product durability published since 1984.

The review encompasses almost four decades of research (1984–2021). Figure 2 reveals an increase in research articles during the last decade, with a notable rise over the previous five years. In addition, three articles from 2022 were included in the selected works since they were already indexed in Scopus at the time of the literature search (December 5, 2021).

**Figure 3:**
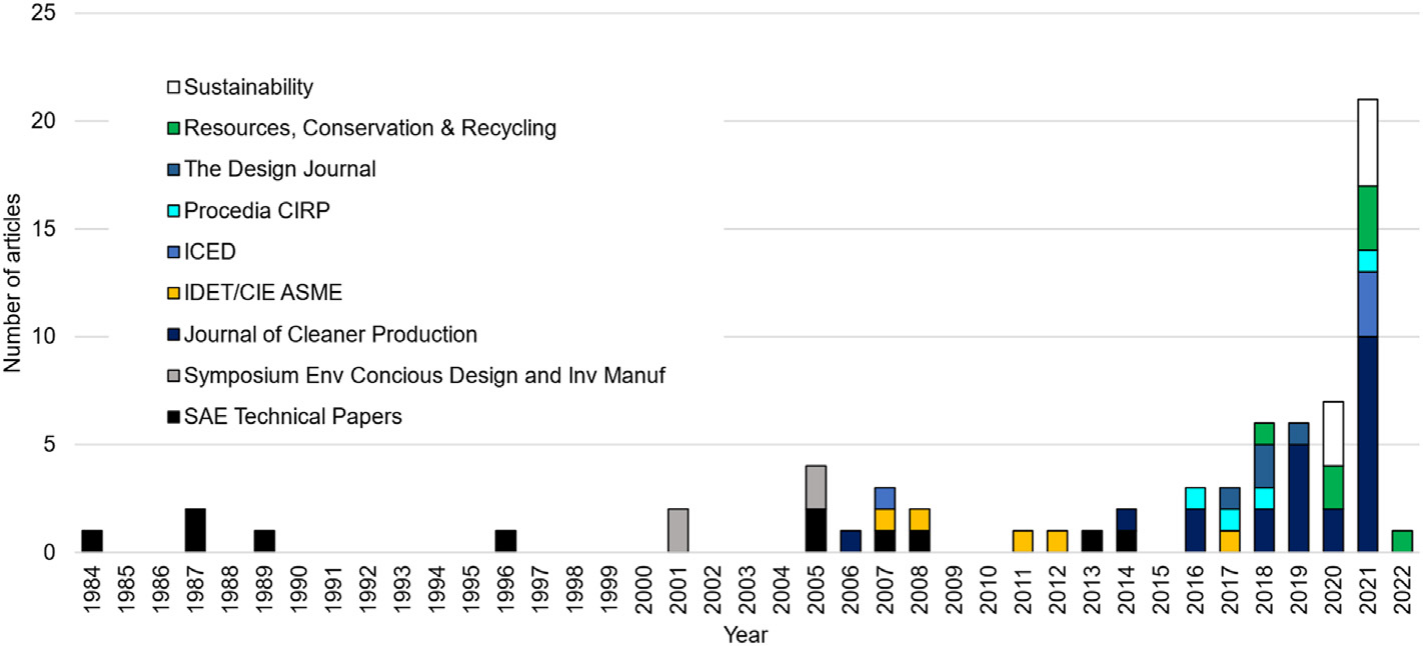
Breakdown of scientific journals and conferences for the final set of eligible papers related to product durability.

Conference papers and journal articles accounted for 22% and 88% of the selected works. Six journals and three conferences contained most of the selected works: *Journal of Cleaner Production* (23); *SAE Technical Papers* (11); *Resources, Conservation & Recycling* (7); *Sustainability* (7); IDET/CIE ASME Conference (5); *Procedia CIRP* (4); *The Design Journal* (4); ICED Conference (4); and The International Symposium on Environmental Conscious Design and Inverse Manufacturing (4). Figure 3 shows the evolution of journals and conferences corresponding to the final set of eligible papers.

### 2.2 Framework for keyword-based and content-based analysis

**Figure 4:**
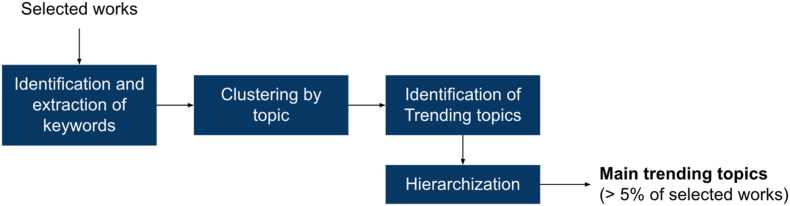
Framework for the keyword-based analysis.

Two primary analyses enabled the identification of research trends, related topics, challenges, and research gaps around the concept of product durability in the product design process. The first corresponds to a keyword-based analysis to identify the main research topics and their implications for product durability (see Figure 4). The keyword-based analysis was performed manually, consolidating all keywords indexed by authors in the 147 selected works. Keywords clusters provide insights about trending topics evolution of main research topics around the theme of product durability; it also enabled the identification of synonyms and relevant words appearing each decade around product durability as a demonstration of the branching process of the theme. This analysis also included a hierarchization process to highlight essential topics based on the frequency of appearance regarding the selected works. The clusters and keywords that were included are shown in Appendix B.

The second is a content-based analysis, which uses a framework based on four main aspects: objectives and methodology; actors involved and lifecycle phases; CE strategies; and design stage, design attributes, and type of products involved (see Table 1). A set of dimensions for analysis arose from reviewing the 147 selected articles according to the four main aspects. The process to define these dimensions consisted of: analysis of existing literature reviews to define dimensions of interest; analysis of selected articles (first round of revision) to define a list of options or alternatives for each dimension; and a refinement process through the second round of revisions to validate and characterize the extracted information.

**Table 1:** Framework for the content-based analysis.

Investigated aspect and relevance	Dimension	Options and definitions
**Objective and methodology** Contrast what previous literature has done and how the objective has been achieved.	Objective	***Propose methodology or guideline:*** proposes or develops methodologies, guidelines, or approaches to determine product durability.
		***Assess properties or parameters:*** proposes an indicator or approach for assessing product durability.
		***Explore or analyze determinants and relationships:*** explores or analyzes stated relationships, hypotheses, or determinants among several variables (*e.g*. consumer behavior and product replacement).
	Method	***Analytical model:*** develops an analytical model with a closed-form solution.
		***Design method:*** proposes a formal method to integrate, measure, or determine product durability. ***Framework:*** proposes a framework related to including and analyzing durability during the product development process.
		***Computer-aided engineering (CAE) simulation:*** develops a model and uses dynamic simulation to solve a problem (system dynamics, finite element analysis).
		***Survey:*** uses a survey to collect empirical data to investigate relationships and implications among variables.
		***Laboratory test:*** reports experiments in a laboratory or controlled environment
**CE strategies** Determination of how CE has been adopted in previous literature.	CE 4R strategy (recirculation of products and parts)	***Reuse:*** addresses the reuse strategy, which implies the recirculation of products. It promotes second-hand products that are still in good condition.
		***Refurbish:*** addresses the refurbish strategy, which involves the ability to repair or re-adjust a product with little time of use.
		***Repair:*** addresses the repair strategy, which involves the ability to replace damaged or non-functional parts to continue using the product.
		***Remanufacture:*** addresses the remanufacturing strategy, which implies repairing and replacing parts after a useful lifecycle to overhaul the product and offer a new one. ***Upgrade:*** addresses the upgrade strategy, which implies modifying the product architecture through modularity to include new components and functionalities to promote extended useful life without product replacement.
**Design stage and design attributes** Identification of design-level approach and the design attributes involved.	Design stage	***Definition of requirements:*** provides valuable data to define product requirements or attributes related to durability, addressed in subsequent design phases.
		***Conceptual design:*** provides a useful approach to define product architecture, functional relationships, and overall constructive design arrangements to promote durability.
		***Basic and detailed design:*** provides a useful approach to define the geometry, materials, and properties that the product must satisfy in terms of durability.
	Design attributes involved	***Material & geometry:*** ***Materials:*** involves durability from material selection for products and parts. ***Geometry:*** involves durability from the definition of geometry for products and parts. ***Other attributes:*** involves durability from other aspects such as functionality or aesthetics.
**Actors involved, lifecycle phase, and type of product** Define the actors addressed by the studies and which lifecycle phases were considered. This aspect also includes the type of product covered by the research.	Actors involved (excluding designer)	***Manufacturer***: addresses implications for the manufacturer of products, components, and materials. ***Retailer:*** addresses implications for the retailer, the actor who sells and distributes products. ***User:*** addresses implications for the final user of the product. ***Service provider:*** addresses implications for the service provider; the actor who offers additional services (maintenance, reuse, remanufacturing). ***End-of-life (EOL) actor:*** addresses implications for the EOL actor, who collects products when they reach EOL.
	Lifecycle phases (excluding the design phase)	***Manufacturing addresses implications for the product production phase (production of components, parts,*** and assembly). ***Sales and distribution:*** addresses implications for the phase where the product is sold and delivered to the final user. ***Usage:*** addresses implications for the phase where the final user uses the product. ***End of use:*** addresses implications for the phase where the product ends its use.
	Type of product	This category includes the type of product analyzed, studied, or covered by the literature (*e.g*. electronics, structural, products in general, automotive).

Note: Investigated aspects and relevance, dimensions, and options were adapted from Bressanelli et al. (2018).

## 3 Results

### 3.1 Keyword-based analysis

After analyzing and clustering the most used keywords in the selected groups, we were able to identify nine topics associated with product durability. Each of these topics is described in the following sub-sections in chronological order.

#### 3.1.1 Fatigue, failure, and reliability

The first approaches found in the literature appeared in the automotive sector (Landgraf, 1987; Landgraf and Conle, 1984; J. Zhou and Goel, 1996), mainly focused on providing highly durable products based on reliability. Research works in this cluster analyzed the response of components and products under mechanical loads, considering stress, deformation, and fatigue (Aldridge, 2004; Baek et al., 1993; Kharul and Pomaje, 1999; Raheja, 2013; Youn et al., 2005), as well as failure modes during the product development process. In addition, some works included modeling and optimization to improve product robustness (Bhide et al., 2005; Choi et al., 2003, 2005; Goel and Singh, 1997; Nolan and Woods, 1989; van den Bogaard et al., 2004; C. J. Wang, 1990). Other studies also included experimental approaches to measure and determine product durability at early design stages (Devlukia and Davies, 1987). Fatigue, failure, and reliability have appeared in recent literature connected with data analytics, accelerated tests, and computer-aided design tools (Burger et al., 2021; Hribersek et al., 2021; Mohammadian and Ait-Kadi, 2010; Munson et al., 2018; Stamminger et al., 2018; Su, 2010; Wei et al., 2012, 2013).

Fatigue, failure, and reliability were essential to product durability at the beginning of the observation period (100% in 1984–1990), which corresponds to the development of the automotive industry and products conceived to last long and provide high reliability and durability. Later, other sustainability-related issues took the attention of researchers (2% during 2016–2020), and interest in this topic seemed to drop. This fact can be explained by the growing relevance of new global issues such as global warming, sustainable development, and environmental issues related to a more conscious use of resources. Nevertheless, the topic continued to develop, with a lower percentual interest, including analysis of sustainability impacts and lifecycle considerations (use of material, repair, remanufacture, upgradability, reconfiguration).

#### 3.1.2 Obsolescence

Obsolescence refers to becoming obsolete and no longer used, even if the product still provides functionality. This topic appeared at the end of the 1990s in the field of electronic parts (Pope et al., 1998; Solomon et al., 2000), and since then, it has had a wide variety of approaches in product design.

Since the early 2000s, designers and engineers responsible for product development have begun to develop methodological approaches to manage obsolescence, with a marked increase during the last decade (Zallio and Berry, 2017). Some approaches have included product upgradability (Umemori et al., 2002; Zhang and Kimura, 2005), product disassemblability (Harmer, 2005), design for lifecycle mismatch (Bradley and Guerrero, 2008), analysis of the product development lifecycle (Brown et al., 2011), lifecycle assessment (Proske and Finkbeiner, 2019), and design for fast consumption and design for restricted technological update (Satyro et al., 2018). As a result, obsolescence is an essential aspect of product design planning (Feldman and Sandborn, 2008; Proske et al., 2016).

Other works have studied consumer perceptions and obsolescence based on product appearance referred to as cosmetic obsolescence (Lilley et al., 2019). Recently, CE strategies have tackled obsolescence through reuse, refurbishment, remanufacture, and upgrade (Barros and Dimla, 2021)In addition, obsolescence has been studied and analyzed to determine the expected life of products and components, mainly supporting businesses centered on purchasing cycles. However, since the last decade, obsolescence has been studied mainly to predict and manage product lifespan and minimize waste generation.

#### 3.1.3 Sustainability and eco-design

This third topic includes environmental impacts from discarding and replacing products, the rapid resource depletion that drives the extraction of new raw materials, and how product durability is relevant to promoting more sustainable consumption (Mont, 2008). The concept of sustainable design, sustainability, design for sustainability, and eco-design started gaining relevance after 2005. In this sense, we were able to identify product design approaches based on remanufacturing (Charter and Gray, 2008), product adaptability (Kasarda et al., 2007), modular upgradability (Agrawal and Ülkü, 2013; J. A. Mesa et al., 2019), and optimization (Kwak and Kim, 2012; Umeda et al., 2007) to define lifetime strategies. Other works have provided eco-design recommendations for manufacturers and consumers related to extending the useful life of products by providing highly durable products or components (Casamayor et al., 2015). Recent studies have also promoted the analysis of multiple lifecycle scenarios, comparing sustainability impacts and the greenness of product lifetime extension (Evrard et al., 2021; Kwak, 2016).

This topic has been boosted since the early 2000s with the predominance of environmental issues, resource depletion, and sustainable development. As a result, the inclusion of sustainability and ecological aspects into product design and development processes has become almost mandatory, and the interest of companies in this topic has increased in the context of reputation and marketing strategies.

#### 3.1.4 Consumer behavior

This topic is one of the most extensive regarding the product durability concept. It includes various approaches covering engineering design, psychology, and marketing. In the early 2000s, research focused on analyzing the consumer behavior that motivates product replacement (Cooper, 2004; van Nes and Cramer, 2005; Woolley, 2003). Other relationships have been studied, such as the influence of the country of design and the country of manufacture on consumer perceptions (Hamzaoui and Merunka, 2006), which affect consumer behavior (Hervé and Mullet, 2009). Product attachment, designed for emotional attachment, is one of the most common fields studied on this topic (Chapman, 2016; Lacey, 2009; Lobos and Babbitt, 2013; Mugge et al., 2010; Page, 2014; van Desmet et al., 2012). Some interesting strategies for product attachment include post-purchase satisfaction (Mugge et al., 2010) and personalization (Fossdal and Berg, 2016).

Product design strategies in this topic target sustainable behavior (Hebrok, 2014), consumption psychology (Xu and Xiong, 2008), promoting more conscious consumer behavior, challenges and strategies for repeat purchase, low-involvement products (Kunamaneni et al., 2019), guidelines for influencing consumer's perceptions of consumer durables through product appearance (Mugge et al., 2018), consumer expectations for product lifetimes (Oguchi et al., 2016), affective design (Agost, 2020), and resilient design (Haug, 2018, 2019). Recent research has determined and analyzed the response of consumers to circular products (reused, refurbished, remanufactured) (Pretner et al., 2021; Sabbaghi and Behdad, 2017; Wallner et al., 2020, 2022). Recently, Bigerna et al. (2021) investigated the degree of willingness of teenagers to pay for products with ecolabels associated with durability attributes. Results showed a preference for repairability as a critical characteristic of circular products.

Consumer behavior initially covered how users behave and desire new products. Nowadays, it covers the emotional and psychological involvement of customers/users regarding a product or brand to promote long useful lifespans and reduce product replacement rates.

#### 3.1.5 Lifecycle assessment/thinking

This topic covers research works oriented toward analyzing relationships between durability and the whole lifecycle. We identified several trends in this topic: lifecycle cost, which focused on calculating the financial cost of durability across the lifecycle of products (Goel and Singh, 1997; Richter et al., 2019; Sandborn et al., 2008); lifecycle assessment, which covers the environmental and economic assessment of the durability of products (Bobba et al., 2016); and lifecycle thinking, which includes product lifecycle strategies (Bauer et al., 2016; Cooper, 2005; Li et al., 2004), analysis of physical and useable life (Koenigsberg et al., 2011), and lifecycle optimization (Yunus et al., 2020).

Nowadays, lifecycle assessment/thinking involves the impact of decisions from early design stages in later stages, such as manufacturing, usage, and end-of-life (EOL). Regarding durability, current analyses include all lifecycle stages from a broader perspective, encompassing business models, lifecycle actors' interaction, and products’ intrinsic value depending on the lifecycle stage.

#### 3.1.6 Product-service systems (PSS)/business models

This topic covers the development of business models based on highly durable products. For example, renting, leasing, and reuse are covered in this topic as strategies to reduce resource consumption and promote material recirculation. Approaches identified were focused on leasing, renting, and remanufacturing (Kerdlap et al., 2021; Mont et al., 2006), servitization (Nazzal et al., 2013), and lifetime extension (Bauer et al., 2016; Ertz et al., 2019; Jensen and Remmen, 2017). In terms of product design, some research works have analyzed CE strategies to promote PSS based on product use and replacement, second-hand use, product discard, and the collection and processing of materials through recycling (Bocken et al., 2016; Franco, 2019; Zeeuw Van Der Laan & Aurisicchio, 2019). CE appears to be a pivotal concept for promoting new business models based on an extended product lifespan and the recirculation of materials (Dahmani et al., 2021). The servitization of products through CE is also a strategy to address product obsolescence (Munten et al., 2021). In recent years, product durability has gained relevance since it supports the development of PSS and business models based on using a product as a service (renting, leasing) and the extended lifespan of products (reuse, refurbish, remanufacturing).

#### 3.1.7 Circular economy (CE)

This is the most cited topic surrounding the product durability concept, especially during the last five years. Almost 50% of selected works mention CE for addressing product durability. CE is also related to lifecycle thinking, PSS, and eco-design. Its main aim is to reduce environmental impacts by promoting new business opportunities and recirculating products, components, and materials.

There are several approaches based on CE strategies that relate to product design. Some salient examples are design for upgradability (Xing and Belusko, 2008), design for remanufacturing and refurbishing (Bakker et al., 2014; Charter and Gray, 2008; Steeneck and Sarin, 2018; W. Wang et al., 2017), design for use optimization (Mont, 2008), design for repairing (Cordella et al., 2021; W. Wang et al., 2017), and design for longevity (Carlsson et al., 2021). Other approaches encompass CE strategies, aiming to provide various alternatives for consideration by designers during early product development phases (Bovea and Pérez-Belis, 2018), while some other approaches focus on measuring and assessing the greenness of extending the lifespan of products (Alfieri et al., 2018; Desing et al., 2021; Franklin-Johnson et al., 2016; Hagejärd et al., 2020). CE has also been analyzed from the design process perspective, considering multiple lifecycles (Asif et al., 2021), product personalization (Maldini et al., 2019), and based on the morphological product structure (Tena, 2021). Similarly, there has been research on indicators to measure product lifespan (Hummen and Desing, 2021), durability tests (Stamminger et al., 2020), and material selection based on durability (J. Mesa et al., 2020).

Interestingly, it was possible to identify the separation between eco-design and circular product design (den Hollander et al., 2017), including well-defined strategies to generate circular flows of products, components, and materials. Furthermore, recent works have focused more on circular product design, for example, the Use2Use design toolkit (Rexfelt, 2021) and the analysis of the barriers to circular product design (J. X. Wang et al., 2022). Nowadays, CE is established as a central theme, and product durability is a key attribute that facilitates several strategies related to the recirculation of products/components (reuse, refurbish, remanufacture, repair, upgrade). Moreover, it is anticipated that CE will attract significant attention in the present decade.

#### 3.1.8 Industry 4.0

This new trending topic is being implemented rapidly in all industries, not just in product development. Furthermore, it is also related to other topics, such as PSS and lifecycle thinking. Regarding product durability, selected works included the use of IoT to support PSS based on circularity (Ingemarsdotter et al., 2020), data analytics to measure and predict economic and ecologically optimal durability of products using data from consumers (Schlegel et al., 2021), and entire frameworks that cover lean design combined with 3D additive manufacturing, mass customization, servitization, and eco-design (Dahmani et al., 2021).

This topic will undoubtedly attract more attention during this decade to analyze, measure, and predict product durability, using digital technologies such as digital twins, additive manufacturing, data-driven design, simulation, and sensors for lifecycle measurement. In addition, personalization and reconfiguration are supported and boosted by Industry 4.0 applications that enable more robust user–product relationships and mitigate rapid product replacement.

#### 3.1.9 Policy/legislation

This last topic emerges as a response of governments in dealing with contamination, climate change, and resource depletion in the world. More durable products enable less resource consumption and become highly relevant in facing global environmental issues. This topic covers the analysis of the impacts of environmental policies based on which firms can adjust product design (Bernard, 2019). In addition, it includes the increase in the adoption of extended producer responsibility (EPR) among companies dedicated to manufacturing and selling tangible products (Alev et al., 2019; Campbell-Johnston et al., 2020) and pre-market producer responsibility (Maitre-Ekern, 2021).

In this topic, it is possible to find research analyzing the implementation and barriers of previous policies implemented in some regions, especially in Europe. Recent studies like the one developed by Hartley et al. (2020), analyzed policy adoption and its success in the European Union. That study used interviews and showed interesting expectations that include more robust and strict standards and norms in production, expansion of circular procurement, tax reliefs, liberalization of waste trading, the use of virtual platforms as key tools, development, and support of eco-industrial parks, and awareness campaigns. Svensson-Hoglund et al. (2021) developed a review that includes the main barriers to implementing repair in the US and EU after implementing some CE policies such as the EU Ecodesign. They highlighted the main challenges for such policies regarding the market governance structure and implications of primarily centralized repair services provided by manufacturers. Similarly, Milios (2021) gathered and analyzed the implementation of CE policies in the Swedish economy, remarking three interesting requirements: (i) public procurement for circular products and services, (ii) increased provision and access to information regarding material flows and circular products, and (iii) government leadership to point out mandatory reuse targets for companies.

This topic is growing, and it is expected that more countries and states will develop more legislation to promote durable products, reduce product replacement, and avoid waste. The consequence will be more service-based than product-based businesses, transferring more responsibility to producers, education focused on promoting sustainable behavior, and CE strategies in domestic and industrial environments.

### 3.2 Evolution of research interest around trending topics

**Figure 5:**
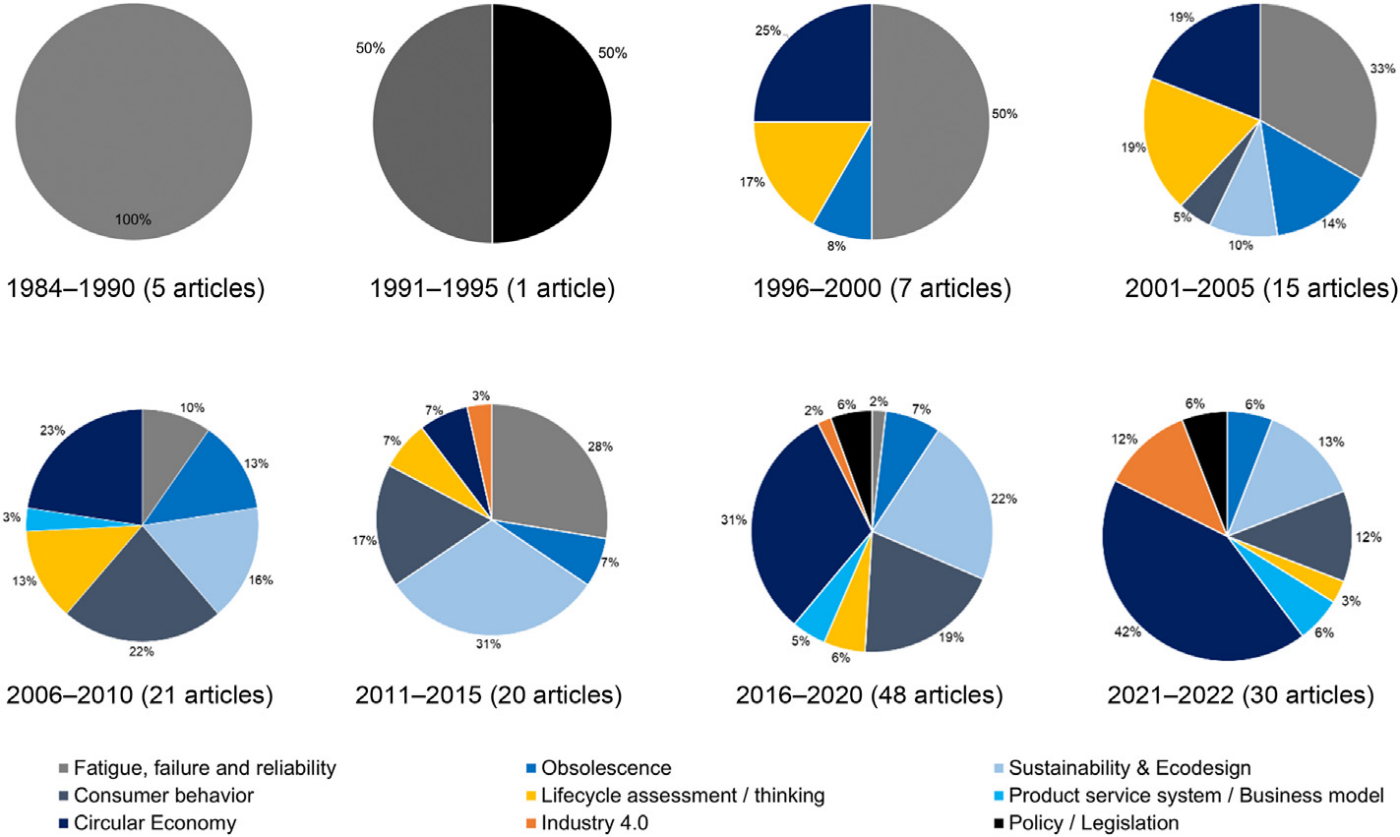
Trending topics from 1984 to 2022 regarding product durability.

Figure 5 summarizes the number of articles and the distribution of the nine trending topics described above. Note that observation time is divided into five-year periods, and the absolute number of articles is different for each period. The last pie chart corresponds to articles published in 2021 (until December 5; some articles from 2022 were also included).

### 3.3 Content-based analysis

This subsection includes the results after analyzing the relationships and distribution of selected works regarding the four dimensions previously established: objective and methodology; CE strategies; the design phase and design attributes; and the actors involved, lifecycle phase, and type of product. The first aspect involved the whole sample (147 articles); meanwhile, the second and third aspects only included articles related to CE (97 articles) and design methodologies (47 articles), respectively. The fourth aspect includes the whole sample as well.

#### 3.3.1 Objective and methodology

In terms of objectives, most of the selected works focused on exploring or analyzing determinants and relationships. The most common methodology was the framework (29 articles), oriented toward studying the concept of product durability from different perspectives, including product conceptualization, manufacture, the fashion industry, and user experience. Other works in the same objective proposed analytical models (13 articles) to measure and determine the influence of design factors (industrial design, aesthetics, perceived value) during the useful life of products. Others developed survey-based studies (16 articles) to identify trends and perceptions about product durability, product replacement motivations, or the hierarchization of design topics based on expert opinion.

The second most relevant objective was proposing a design methodology or guidelines. Research works with this objective were associated with product design activities, methods, and approaches to defining product attributes, including product durability. These works mainly focused on defining materials and geometry to achieve reliable and durable products. Most articles with this objective corresponded to analytical models and design methods (15 articles for each one), followed by 11 articles classified as frameworks oriented toward design, three articles that employed computer-aided engineering (CAE) simulation, two articles based on a review or previous methodologies, and one article based on expert opinion through surveys.

**Figure 6:**
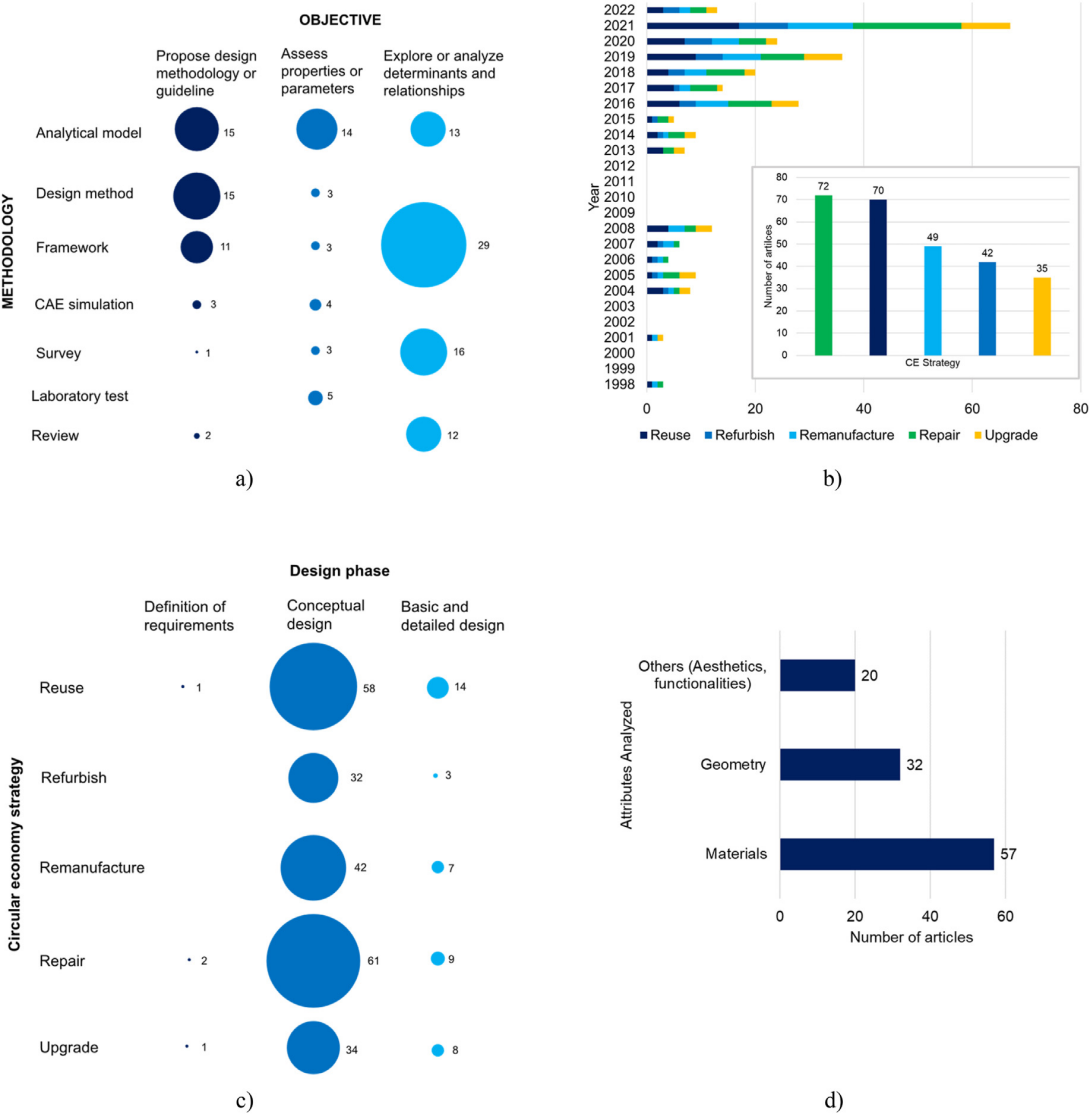
(a) Objective vs. methodology relationships among selected works. (b) Breakdown of CE approaches found in selected works (97 of 147 articles). The column chart shows the distribution of articles regarding CE strategies, while the bar chart illustrates the evolution of these strategies over the last 40 years. (c) Design phase vs. CE strategy relationships among selected works. This analysis only includes 48 articles related to methodological design approaches from the selected works that also involve CE strategies. (d) Attributes analyzed in selected works regarding product durability. Only 78 selected works mentioned the influence and determination of durability from such attributes.

Finally, the least found objective was assessing properties and parameters. This objective included articles dedicated to measuring or quantifying parameters related to product durability. It also included the development of indicators or metrics related to durability. Within this objective, most research efforts were oriented toward analytical models (14 articles), followed by laboratory tests and CAE simulation (five and four articles, respectively), and design methods, frameworks, and surveys (three articles for each). Figure 6a illustrates the results for this dimension after analyzing the articles from selected works.

#### 3.3.2 Circular economy (CE) strategies

After analyzing the selected works, we identified 97 articles related to CE strategies. These articles represent 66% of the study sample. The analysis of this aspect was limited to articles including or mentioning CE strategies related to product durability (reuse, repair, refurbish, or remanufacture). Of the 97 articles, 74% focused on repairing products, 72% on reusing, and 50% and 43% on remanufacturing and refurbishing, respectively. In addition, the selected works also covered upgrading (36% of articles). Note that an article can be included in more than one strategy.

Articles mentioning CE strategies appeared during the first decade of the 21st century, and the CE concept formally appeared after 2010 in the selected works. 2021 featured a 140% increase in articles addressing CE and product durability compared to 2020. Figure 6b shows the breakdown of articles that include CE and the evolution over time in the number of articles.

#### 3.3.3 Design phase and design attributes

A total of 47 articles proposed a design methodology or guidelines. We identified the design phase in which the approach can be applied, considering the CE strategy. Accordingly, most works were on the conceptual design, followed by basic and detailed design, with only a few addressing the definitions of requirements. Articles on the conceptual design phase were oriented toward repair, reuse and remanufacture of components (61, 58, and 42, respectively), followed by the upgrade (34 articles), and refurbish (32 articles). In the basic and detailed design phase, most articles were classified in the reuse, repair, upgrade, and remanufacture categories (14, nine, eight, and seven, respectively), with only three articles in refurbish. Figure 6c shows the distribution of works in terms of the design phases.

Finally, 78 works covered attributes from the design stage regarding engineering attributes in the selected works. Among these, 57 focused on materials, followed by geometry (32) and aesthetics/functionality (20).

#### 3.3.4 Actors involved, lifecycle phase, and type of product

After analyzing the selected works, it was found that the most relevant actor was the producer; most articles covered the product design providing methodological tools and guidelines that the producer could implement. A few articles included the user, service provider, and EOL actor, while only a few articles included the retailer. The most covered lifecycle phase was usage, including psychological approaches, emotive design, and consumer behavior. Manufacturing closely followed usage, covering durability through materials, geometry, and transformation processes to obtain finished products. The EOL phase was only moderately covered in the selected works; finally, sales and distribution were only marginally studied.

**Figure 7:**
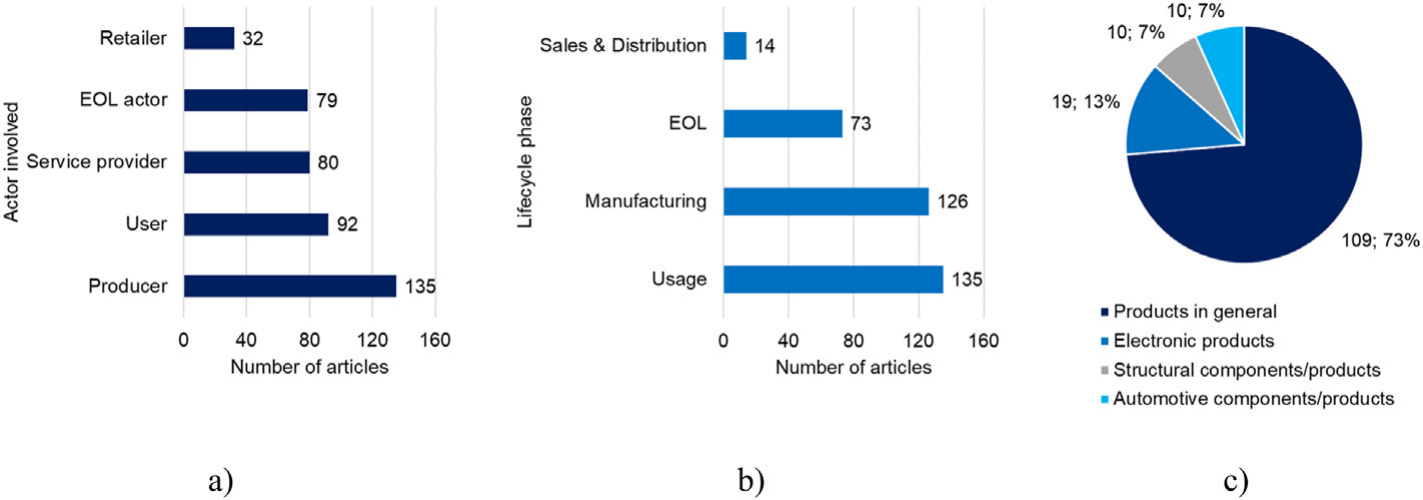
(a) Actors involved in product durability in selected works. (b) Lifecycle phases considered in selected works. (c) Type of product analyzed in selected works.

Concerning the type of products studied in selected works as case studies, it was identified that almost three-quarters of papers were not designed to cover a specific type of product. As a result, only three types of products could be identified, covering a quarter of selected works: electronics, structural, and automotive. Figure 7 summarizes the results of this research aspect.

## 4 Discussion: trending topics, gaps, and research agenda for product durability and the circular economy (CE)

### 4.1 Trending topics

After studying the nine trending topics identified in the research literature throughout the keyword-based analysis, the following issues can be highlighted as design considerations for product durability:

• 2004 spike in Figure 2 is consistent with a disruptive moment in which product lifespan and therefore durability awakes the interest of researchers, policymakers, and practitioners as a response to a global concern related to product obsolescence and its impact on sustainability. As a cornerstone, in 2002, European Union proposed its Waste from Electrical and Electronic Equipment WEEE directive (2002/96/EC) as a first step in measuring and acting on recycling, reuse, and management of materials derived from electronic and electrical devices to avoid contamination. In terms of research, Cooper (Cooper, 2004, 2005) stands out as one of the most relevant authors regarding obsolescence and product lifespan analysis. He proposed the concept of “throwaway society”, highlighting the unsustainable consumption patterns existing during the early 2000s and the need for industrialized nations to consider sustainability in their product development processes. These first contributions initiated a growing research field covering consumer behavior, aesthetics in product lifespan, marketing, product-service systems, and public policies.
• From Figure 3, the evolution in the scope of journals from 1984 to the present is noticeable, passing from technical journals dedicated to fatigue and reliability to others focused on more environmental issues, such as resource consumption, environmental impacts, waste management, and consumer behavior drivers. This outcome is also evident in the topic “Sustainability and eco-design,” which has been an ever-present topic since the early 2000s in designing products considering durability.
• The “Fatigue, failure, and reliability” topic and other related design aspects were the most studied during the first ten years covered by this review. However, interest in this topic has dropped dramatically during the last five years, representing only 2% of all articles analyzed.
• “Obsolescence” has appeared as a relevant topic since the end of the 1990s. Most researchers addressed this topic as a non-desired attribute of products, mainly when used to accelerate product replacement and the desire for product changes, even when the product is functional. However, obsolescence can be studied to promote “PSS/business models” based on product acquisition, extended lifespan, servitization, and product leasing. This trend in servitization is also boosted by digitalization, demonstrating how needs such as housing, transportation, and alimentation are based on apps and web services.
• The “Circular economy (CE)” topic has gained high relevance during the last decade, making it an essential topic for product durability. Therefore, research efforts must demonstrate effectiveness and sustainability benefits to guarantee a successful implementation in the industrial field with business models supported by servitization and CE strategies (reuse, refurbish, remanufacture and repair).
• *“*Consumer behavior” is a crucial aspect that must be addressed through design to ensure wider acceptance of circular products (reused, remanufactured, refurbished), encompassing emotional design, product attachment, and more conscious consumption patterns.
• From early design phases, it is possible to define the sustainability impact of products and components; therefore, using “Lifecycle assessment/thinking” tools is relevant to predicting and adjusting design attributes. Furthermore, since durability is an engineering aspect for manufacturing, usage, and EOL, measuring and considering the whole lifecycle of a product or component from a holistic perspective is necessary.
• “Industry 4.0” has become an emerging topic in the last decade. It can help improve product performance in terms of durability. Tools such as digital twins, CAE simulation, cloud computing, and lifecycle monitoring enable more robust analysis and rapid improvement cycles from the early design stages.
• “Policy/legislation” is a topic that has accompanied the growing trend of CE and sustainability in recent years. More governments and states are aware of the necessity for more policies concerning producers, users, and EOL actors' responsibility to promote CE on a large scale. In addition, waste generation is a global concern, and more actors are conscious of the potential value of materials, components, and products in CE models.

### 4.2 Content-based analysis

We examined the results of the content-based analysis to identify gaps and propose a research agenda for advancing the research of product durability (summarized in Table 2).

**Table 2:** Research gaps and research agenda regarding product durability.

Aspect	Research gap	Research agenda
Objective and methodology	Most research is focused on framework studies, with little attention paid to design methods and methodological tools to measure, determine, and predict product durability. Durability is not studied as an engineering parameter in design approaches, and only a few articles have proposed CAE simulations and laboratory tests. There is very little research on indicators to measure product durability, primarily to be implemented in a CE environment.	I) Develop more design methods and indicators to measure, determine, and predict product durability in different lifecycle scenarios. II) Develop more approaches based on CAE simulations and laboratory tests. III) Develop indicators for product and component durability based on CE strategies
CE strategies	Most works are oriented toward single CE strategies instead of a broader scope that covers several strategies simultaneously (only 14% of selected articles include all CE strategies). Refurbish is less studied compared to reuse, remanufacture, and repair. There is a lack of research approaches covering all CE strategies and guidelines to re-design products depending on the strategy's benefits and limitations. There is a need for decision-making research to choose and hierarchy CE strategies considering product durability and the best business model.	IV) Target more approaches that include all CE strategies simultaneously. V) Increase research efforts in refurbishment. VI) Explore how all CE strategies can be integrated into design guidelines for the re-design of products. VII) Develop decision-making approaches to select and compare CE strategies based on the product durability and the preferable business models.
Design stage and design attributes	Research has paid little attention to the definition of requirements in the product design process. There is a need for more product design research on basic and detailed design. Research has paid little attention to the definition of materials and geometry as primary parameters that define durability during the product design process.	VIII) Develop more approaches focused on determining the definition of requirements, including product durability and basic and detailed design. IX) Develop approaches oriented toward defining/selecting geometry and materials to achieve suitable product durability from early design phases.
Involved actors, lifecycle phase, and type of product	Research has paid little attention to retailers. Only a few articles have included implications for the stage of sales and distribution. Most research has been focused on products in general; there is a generalization of approaches, and only a few are specified around specific types of products.	X) Focus more research on retailers and the sales and distribution stage. XI) More research is required for specific products, identifying singularities in the lifecycle (*i.e*. smartphones, home appliances, structural components, furniture, clothing).

The analysis of the objectives and methodologies of the selected works was focused on what has been proposed in the previous literature and how the objectives were achieved. We found that most research efforts have been dedicated to explorative and analytical approaches, followed by methodological and assessment approaches. In explorative works, the most common methodology was the framework. Consequently, a first research direction involves developing more methodological approaches and assessment tools to define, measure, and predict product durability considering different lifecycle scenarios (*i.e*. products’ extended lifespan through reuse or servitization, product repairability, or upgrading). The highlighted research direction points out new useful design frameworks, practical methods, indicators, and lifecycle assessment tools. The purpose is to consider durability from early design stages, based on quantitative analysis, decision-making tools, and lifecycle impacts associated with the durability of products and associated services.

Despite diverse methodologies, a gap is identified in developing CAE simulations and laboratory tests, where research contribution is lower compared to other methodological approaches. Thus, another research direction involves defining new methods and procedures using simulation and laboratory tests to measure product/component durability. Here, using Industry 4.0 tools, such as digital twins, provides significant benefits since it is possible to analyze the whole lifecycle of products without prototyping or applying destructive tests. Digitalization is critical in reducing prototyping and testing costs and ensuring durability, thus providing enough lifespan extension according to the company's interest and design strategy. As a major advantage, digital twins provide a robust approach to testing unlimited lifecycle scenarios with different use conditions and patterns that serve as input to determine the best durability performance for any product or system.

Similarly, another gap is found in developing indicators to measure and assess product durability, especially for applying CE strategies (reuse, refurbish, remanufacture, repair). Consequently, another research direction involves developing indicators and metrics for component or product durability based on engineering properties. This research direction uses the mechanical and durability properties of materials and geometrical attributes not only for conventional resistance calculations but also for generating assessment approaches for material and manufacturing processes selection for durable products. Such a selection should contemplate future reuse, repair, refurbish and remanufacture scenarios of parts or even the whole product.

Only 14% of works related to CE included an overall perspective, with most focusing on one or two strategies. Therefore, there is a need to explore and generate approaches that include all CE strategies instead of isolated strategies. For example, highly durable products enable the reuse (exchange among users in different useful cycles), repair, and replacement of spare parts to support refurbishment and remanufacture. Another gap identified is related to refurbishment, which is less studied than other CE strategies since it has limitations in logistic processes to share and offer second-hand products and the need for most robust and effective campaigns from manufacturers that have more resources (user data and customer behavior patterns) to collect products, parts and generate markets based on refurbishment with warranties and confidence among customers or users.

Another gap concerns the design guidelines for re-designing products adopting CE strategies. A promising research avenue regarding this topic implies the development of formal methods and methodological contributions to include reuse, refurbish, remanufacture, and repair from early design stages, promoting less product replacement and high reliability to avoid discarding components. Finally, the last gap in this respect is the lack of decision-making approaches to identify and select the most suitable CE strategies and business models depending on the product. Therefore, another research direction must facilitate the comparison of strategies and their potential business models for designers, practitioners, product planners, and producers. Durability needs to be studied and implemented according to each product type to generate profitability and less environmental footprint. In some cases, it is better to remanufacture instead of recycling (which implies resource consumption in terms of energy, water, and consumables, among others), and some products are more likely to be reused than others (i.e., medical instruments that need to be disposed of after a single use).

Regarding the design stage, most works have been oriented toward conceptual design activities. There is a gap in research on basic and detailed design, especially on the requirements definition. Thus, research should address the lack of methodological approaches for determining durability in basic and detailed design, where materials and geometry are established based on failure criteria calculations and systematic processes. Similarly, researchers should pay attention to the definition of requirements, where initial engineering specifications are settled. Such specifications can also include durability parameters. In this respect, the second research direction in this context is the need for procedures to formally define or select geometry and materials, which are essential attributes for product durability. The definition of product architecture and parts need to be developed considering which ones can be reused, repaired, refurbished, or remanufactured. The selection of geometry also implies the selection of reversible joints to facilitate future upgrading processes and easiness for repair cycles. The selection of materials with high resistance to environmental conditions and wear is critical to extending the lifespan of products and parts. Thus, the durability of the whole product depends on the durability of the less durable component.

Two main research gaps were identified regarding the actors involved. First, most works focused on producers, users, and EOL actors. Consequently, one research direction is to develop research that includes actors in logistic and supply chain processes. For example, retailers are responsible since they have a direct relationship with customers and users. Consequently, retailers are key actors for returning products and components and can serve as a collection center to promote the recirculation of products (reuse, refurbish, remanufacture, repair). Second, most research was generally oriented toward products (no type of product specification). However, there are differences in durability in the context of, for example, electronics, structural engineering, domestic appliances, furniture, and medical devices. Therefore, it is necessary to generate further research approaches for all products with singularities in manufacturing, use, and final disposal.

As an additional finding, in the analysis of selected works, authors do not formally address social issues associated with product durability. However, high durable products can affect employees related to manufacturing, distribution, and selling; since the replacement rate of products decreases and users keep products for more extended periods. As mentioned by Repp et al. (Repp et al., 2021) CE transition could lead to a decrease in employment in low to upper-middle-income countries focused on labor-intense apparel production. Therefore, it is necessary to compensate employment with jobs related to extended lifespan products such as reuse, repair, refurbish and remanufacture. Policymakers and governments are also responsible for compensating for that potential situation and include proper ethical considerations during the enforcement of the CE transition. The concept of Circular Justice proposed by Repp et al. is an interesting approach to facing such potential harmful consequences of CE in low and upper-medium economies.

### 4.3 Barriers to durable products

Success for achieving a massive extension of product lifespan through durability depends on social, economic, environmental, and technical barriers that vary from one country or region to another depending on socio-cultural, economic, environmental, and type of industry. However, several barriers are shared across different geographic regions and industrial sectors. This subsection summarizes barriers to implementing CE strategies around product lifespan extension, focusing on those more related to product durability.

Concerning electrical and electronic equipment, it is remarkable the lack of unified EPR policies addressing issues from e-waste management, higher costs of CE processes compared to linear ones (for instance, acquisition of spare parts and repair processes), lack of financial resources for CE investments, and lack of acceptance or interest in CE practices in consumers (Rizos and Bryhn, 2022; Wrålsen et al., 2021). The lack of government incentives in manufacturing industries is also a critical issue. Other critical issues are the unavailability of appropriate partners for establishing industrial ecosystems and circular materials flows, the low cost of virgin materials compared to recycled materials, and poor attention to the end-of-life phase in product design (Kumar et al., 2019). In terms of supply chain structures, there is not a standard system for performance indicators to measure CE in the supply chain, difficulties in establishing the correct price of circular products, high cost of environmentally friendly suppliers, lack of skills related to CE in employees, and lack of reliable public information to facilitate the acquisition of reused or remanufactured products (Govindan and Hasanagic, 2018). Other sectors like the textile industry face several challenges: high cost for producing and selling circular products, lack of consumer awareness and interest, operation of manufacturing processes is still under a linear pattern, limited circular designs, high upfront investment cost, and the delivery of high-quality circular products (Hartley et al., 2022).

Several barriers are common to any type of tangible product regarding the engineering design process: limited product design for easy disassembly, repair, refurbishment, and remanufacturing; lack of advanced knowledge of materials and chemical properties, lack of product standards for CE, and widespread and socially accepted planned obsolescence in products (Milios, 2021).

Similarly, regarding repairability, several barriers are also common: the negative stigma attached to repaired products, lack of technical knowledge to repair with high quality and to provide robust lifespan extension, and design-related problems associated with geometry and materials considerations during early product embodiment stages (Nazlı, 2021).

### 4.4 Limitations of the study

Four main limitations can be highlighted in this literature review study:

• Gray literature was not included to ensure all the content analyzed corresponded to peer-review processes. Thus, it ensures more scientific rigor and transparency for readers interested in product durability and engineering design. Therefore, this study did not include some approaches and technical information like reports, independent research projects, and legislative-related documentation.
• It is possible that some of the newest literature was not included since a significant amount of time was employed during the data analysis and review processes.
• Although the main research topics were identified after analyzing selected works, the analysis and discussion of such topics are bounded to the field of product design. However, a complete review analysis can be developed for each topic more profoundly, including other considerations such as the evolution of legislative projects, marketing strategies, and public-private initiatives around the concept of product durability.
• The analysis and discussion of results in this article were developed using an engineering standpoint. Nevertheless, it is remarkable that the concept of product durability requires a multidisciplinary perspective that includes other research areas in psychology, consumer behavior, product aesthetics, and even the fashion industry. Moreover, some topics are susceptible to be studied in detail outside the engineering viewpoint or combining research efforts of multidisciplinary teams.”

## 5 Conclusion

This article contributes to the analysis and inclusion of durability into the product design process in light of the CE concept. A total of 148 articles were systematically selected and analyzed following the methodology and frameworks described in Section 2.

By analyzing the selected works, this paper has discussed and shed light on: (i) trending topics related to product durability during the last four decades; (ii) what previous literature has done and how the objectives have been achieved; (iii) how CE strategies have been adopted in previous literature; (iv) the identification of a design-level approach and the design attributes involved; and (v) which actors, lifecycle phases, and type of products have been considered. Addressing these elements has contributed to filling the research gaps in product durability and its further application in the industry. The contributions of this review are the analysis of trending topics and the research agenda presented in Section 4. In addition, the research agenda highlights research directions for scholars interested in continuing the study of product durability within the CE concept. Some studies, such as the one of Kirchherr and Santen (Kirchherr and van Santen, 2019), point out the necessity of conducting studies that help practitioners understand how CE can be implemented rather than providing definitions and conceptualizations. In this sense, we point out that from a practical perspective, this systematic review provides a means for producers, service providers, EOL actors, and policymakers to use the evidence of previous research to inform their decisions. Specifically, they can be informed by the findings from extant research at the intersection of product durability and CE.

Finally, this study has its limitations in that the systematization of the information is affected by researcher bias. Section 2 details the methodology adopted to mitigate this issue as far as possible. Another limitation is examining only scientific journal articles, excluding other literature (gray literature, books, technical reports from organizations) that may offer significant contributions.

## Declarations

### Author contribution statement

All authors listed have significantly contributed to the development and the writing of this article.

### Funding statement

This work was supported by Vicerrectoría de Investigación, Creación e Innovación Universidad del Norte.

### Data availability statement

Jaime, A. Mesa was supported by Vicerrectoría de Investigación, Creación e Innovación Universidad del Norte.

### Declaration of interest's statement

The authors declare no conflict of interest.

### Additional information

No additional information is available for this paper.

## Figures and Tables

**Figure:**
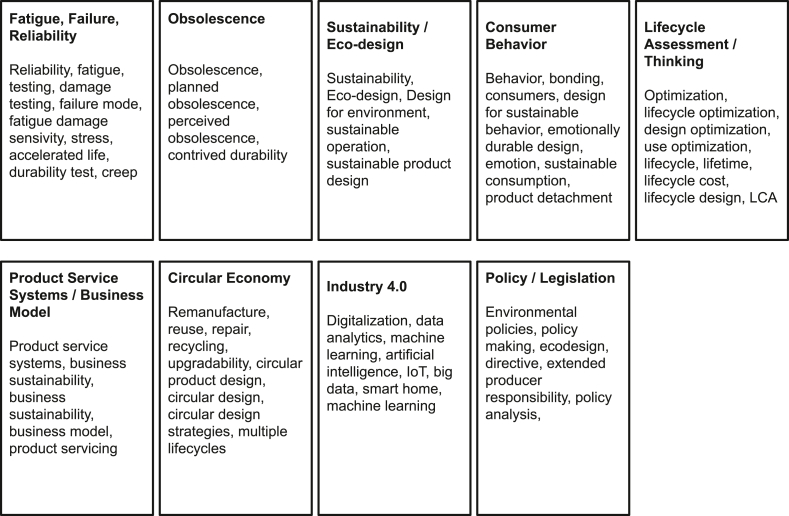


## Appendix A Search query

(TITLE-ABS-KEY (product AND (design AND ((exten∗ AND (lifetime OR lifetime)) OR durab∗ OR obsole∗)) OR longevity OR “circular economy” OR circularity) AND NOT TITLE-ABS-KEY ((concrete OR building OR cement∗ OR corrosi∗ OR cataly∗ OR electrode OR immune∗ OR medic∗ OR fission OR adhesive OR fish∗ OR sleep∗ OR quarry OR asphalt OR pavement OR vineyard OR briquette OR pellet OR cellulose OR scaffolds OR cogniti∗ OR tumor∗)) AND NOT TITLE-ABS-KEY ((tumor∗ OR sensor OR rfid OR chromatograp∗ OR coating OR tribolog∗ OR filament OR dengue OR zeolit∗ OR kaolin OR touris∗ OR roof OR cathode OR anode OR crop OR vaccine OR drug OR “wood pulp” OR timber OR disorder OR coast∗ OR “wind tunnel” OR fender)) AND NOT TITLE-ABS-KEY ((baker∗ OR farmer OR harvest∗ OR flavonoid OR tobacco OR oilfield OR cattle OR cancer OR carcinogenic OR bioremediation OR uranium OR “dairy products” OR dft OR mortgage OR patient OR mortality OR granadilla OR bacteria OR soil OR fertilizer OR seed)) AND NOT TITLE-ABS-KEY ((drosophila OR indigenization OR muta∗))) AND (LIMIT-TO (SRCTYPE, “j”) OR LIMIT-TO (SRCTYPE, “p”)) AND (LIMIT-TO (DOCTYPE, “ar”) OR LIMIT-TO (DOCTYPE, “cp”) OR LIMIT-TO (DOCTYPE, “re”)) AND (LIMIT-TO (LANGUAGE, “English”)).

## References

[bib1] Agost M. (2020).

[bib2] Agrawal V.v., Ülkü S. (2013). The role of modular upgradability as a green design strategy. Manuf. Serv. Oper. Manag..

[bib3] Aguiar M.F., Mesa J.A., Jugend D., Pinheiro M.A.P., Fiorini P.D.C. (2022). Circular product design: strategies, challenges and relationships with new product development. Manag. Environ. Qual. Int. J..

[bib4] Aldridge D.S. (2004). A general process for defining product durability requirements. J. IEST.

[bib5] Alev I., Agrawal V., Atasu A. (2019). Extended producer responsibility for durable products. SSRN Electron. J..

[bib6] Alfieri F., Cordella M., Sanfelix J., Dodd N. (2018). An approach to the assessment of durability of energy-related products. Procedia CIRP.

[bib7] Alhawari O., Awan U., Bhutta M.K.S., Ali Ülkü M. (2021). Insights from circular economy literature: a review of extant definitions and unravelling paths to future research. Sustainability.

[bib8] Asif F.M.A., Roci M., Lieder M., Rashid A. (2021).

[bib9] Baek W.K., Stephens R.I., Dopker B. (1993). Integrated computational durability analysis. Journal of Manufacturing Science and Engineering, Transactions of the ASME.

[bib10] Bakker C., Wang F., Huisman J., Hollander M. den. (2014). Products that go round: exploring product life extension through design. J. Clean. Prod..

[bib11] Barros M., Dimla E. (2021).

[bib12] Bauer T., Mandil G., Naveaux É., Zwolinski P. (2016). Lifespan extension for environmental benefits: a new concept of products with several distinct usage phases. Procedia CIRP.

[bib13] Bernard S. (2019). Multidimensional green product design. Environ. Resour. Econ..

[bib14] Bhide G.D., Chavan S.P., Sobale A. (2005). CAE used for durability analysis - a case study. SAE Technical Papers.

[bib15] Bigerna S., Micheli S., Polinori P. (2021). New generation acceptability towards durability and repairability of products : circular economy in the era of the 4th industrial revolution. Technol. Forecast. Soc. Change.

[bib16] Bobba S., Ardente F., Mathieux F. (2016). Environmental and economic assessment of durability of energy-using products : method and application to a case-study vacuum cleaner. J. Clean. Prod..

[bib17] Bocken N.M.P., de Pauw I., Bakker C., van der Grinten B. (2016). Product design and business model strategies for a circular economy. Journal of Industrial and Production Engineering.

[bib18] Boulos S., Sousanoglou A., Evans L., Lee J., King N., Facheris C., Donelli M. (2015).

[bib19] Bovea M.D., Pérez-Belis V. (2018). Identifying design guidelines to meet the circular economy principles: a case study on electric and electronic equipment. J. Environ. Manag..

[bib20] Bradley J.R., Guerrero H.H. (2008). Product design for life-cycle mismatch. Prod. Oper. Manag..

[bib21] Bressanelli G., Adrodegari F., Perona M., Saccani N. (2018). Exploring how usage-focused business models enable circular economy through digital technologies. Sustainability.

[bib22] Brown T.E., Bartholomew S.E., Smykowski A.C., Carlson C.F. (2011). Proceedings of the ASME 2011 International Design Engineering Technical Conferences & Computers and Information in Engineering Conference.

[bib23] Bundgaard A., Mosgaard M., Remmen A. (2017). From energy efficiency towards resource efficiency within the Ecodesign Directive. J. Clean. Prod..

[bib24] Burger M., Dre K., Speckert M. (2021).

[bib25] Campbell-Johnston K., Calisto Friant M., Thapa K., Lakerveld D., Vermeulen W.J.V. (2020). How circular is your tyre: experiences with extended producer responsibility from a circular economy perspective. J. Clean. Prod..

[bib122] Carlsson S., Mallalieu A., Almefelt L., Malmqvist J. (2021). Design for longevity - a framework to support the designing of a product’s optimal lifetime. International Conference on Engineering Design.

[bib26] Casamayor J., Su D., Sarshar M. (2015). Extending the lifespan of LED-lighting products. Architect. Eng. Des. Manag..

[bib27] Chapman J. (2016). Proceedings - D and E 2016: 10th International Conference on Design and Emotion - Celebration and Contemplation.

[bib28] Charter M., Gray C. (2008). Remanufacturing and product design. Int. J. Prod. Dev..

[bib29] Choi K.K., Tang J., Hardee E., Youn B.D. (2005). Application of reliability-based design optimization to durability of military vehicles. SAE Technical Papers.

[bib30] Choi K.K., Youn B.D., Tang J. (2003). Proceedings of DETC’03 ASME 2003 Design Engineering Technical Conferences and Computers and Information in Engineering Conference.

[bib31] Cooper T. (2004). Inadequate Life?Evidence of consumer attitudes to product obsolescence. J. Consum. Pol..

[bib32] Cooper T. (2005). Slower consumption. Reflection on product life spans and the “throwaway society. J. Ind. Ecol..

[bib33] Cordella M., Al F., Clemm C., Berwald A. (2021). Durability of smartphones : a technical analysis of reliability and repairability aspects. J. Clean. Prod..

[bib34] Cramer J. (2014).

[bib35] Dahmani N., Benhida K., Belhadi A., Kamble S., Elfezazi S. (2021). Smart circular product design strategies towards eco-effective production systems : a lean eco-design industry 4 . 0 framework. J. Clean. Prod..

[bib36] den Hollander M.C., Bakker C.A., Hultink E.J. (2017). Product design in a circular economy: development of a typology of key concepts and terms. J. Ind. Ecol..

[bib37] Desing H., Braun G., Hischier R. (2021). Resource pressure – a circular design method. Resour. Conserv. Recycl..

[bib38] Devlukia J., Davies J. (1987).

[bib39] Ellen MacArthur Foundation (2015).

[bib40] Ertz M., Leblanc-Proulx S., Sarigöllü E., Morin V. (2019). Made to break? A taxonomy of business models on product lifetime extension. J. Clean. Prod..

[bib41] European Commission (2020). https://eur-lex.europa.eu/legal-content/EN/TXT/?qid=1583933814386&amp;uri=COM:2020:98:FIN.

[bib42] European Parliament, C. of the E. U. (2009). https://eur-lex.europa.eu/legal-content/EN/ALL/?uri=celex:32009L0125.

[bib43] Evrard D., Rejeb H. ben, Zwolinski P., Brissaud D. (2021).

[bib44] Feldman K., Sandborn P. (2008). 2007 Proceedings of the ASME International Design Engineering Technical Conferences and Computers and Information in Engineering Conference, DETC2007, 4(Gaines 1991).

[bib45] Fiksel J.R. (2009). Design for environment: a guide to sustainable product development. Sustain. Dev..

[bib46] Fontana A., Barni A., Leone D., Spirito M., Tringale A., Ferraris M., Reis J., Goncalves G. (2021). Circular economy strategies for equipment lifetime extension: a systematic review. Sustainability.

[bib47] Fossdal M., Berg A. (2016). Proceedings of the 18th International Conference on Engineering and Product Design Education: Design Education: Collaboration and Cross-Disciplinarity, E and PDE 2016, September, 95–100.

[bib48] Franco M.A. (2019). A system dynamics approach to product design and business model strategies for the circular economy. J. Clean. Prod..

[bib49] Franklin-Johnson E., Figge F., Canning L. (2016). Resource duration as a managerial indicator for Circular Economy performance. J. Clean. Prod..

[bib50] Goel P.S., Singh N. (1997). A framework for integrating quality, reliability, and durability in product design with life-cycle cost considerations. Qual. Eng..

[bib51] Govindan K., Hasanagic M. (2018). A systematic review on drivers, barriers, and practices towards circular economy: a supply chain perspective. Int. J. Prod. Res..

[bib52] Gümüş M., Ray S., Yin S. (2013). Returns policies between channel partners for durable products. Market. Sci..

[bib53] Hagejärd S., Ollár A., Femenías P., Rahe U. (2020). Designing for circularity-addressing product design, consumption practices and resource flows in domestic Kitchens. Sustainability.

[bib54] Haines-Gadd M., Chapman J., Lloyd P., Mason J., Aliakseyeu D. (2018). Emotional durability design Nine-A tool for product longevity. Sustainability.

[bib55] Hamzaoui L., Merunka D. (2006). The impact of country of design and country of manufacture on consumer perceptions of bi-national products’ quality: an empirical model based on the concept of fit. J. Consum. Market..

[bib56] Harmer K. (2005). Proceedings - Fourth International Symposium on Environmentally Conscious Design and Inverse Manufacturing, Eco Design 2005.

[bib57] Hartley K., Roosendaal J., Kirchherr J. (2022). Barriers to the circular economy: the case of the Dutch technical and interior textiles industries. J. Ind. Ecol..

[bib58] Hartley K., van Santen R., Kirchherr J. (2020). Policies for transitioning towards a circular economy: expectations from the European Union (EU). Resour. Conserv. Recycl..

[bib59] Haug A. (2018). Defining ‘resilient design’ in the context of consumer products. Des. J..

[bib60] Haug A. (2019). Psychologically durable design–definitions and approaches. Des. J..

[bib61] Hebrok M. (2014). Design for longevity: taking both the material and social aspects of product-life into account. J. Des. Res..

[bib62] Hervé C., Mullet E. (2009). Age and factors influencing consumer behaviour. Int. J. Consum. Stud..

[bib63] Hribersek M., Erjavec M., Hlebanja G., Kulovec S. (2021). Durability testing and characterization of POM gears. Eng. Fail. Anal..

[bib64] Hummen T., Desing H. (2021). Resources , Conservation & Recycling When to replace products with which (circular) strategy ? An optimization approach and lifespan indicator. Resour. Conserv. Recycl..

[bib65] Ingemarsdotter E., Jamsin E., Balkenende R. (2020). Opportunities and challenges in IoT-enabled circular business model implementation – a case study. Resour. Conserv. Recycl..

[bib66] Jensen J.P., Remmen A. (2017). Enabling circular economy through product stewardship. Procedia Manuf..

[bib67] Kasarda M.E., Terpenny J.P., Inman D., Precoda K.R., Jelesko J., Sahin A., Park J. (2007). Design for adaptability (DFAD)-a new concept for achieving sustainable design. Robot. Comput. Integrated Manuf..

[bib68] Kerdlap P., Gheewala S.H., Ramakrishna S. (2021). To rent or not to rent : a question of circular prams from a life cycle perspective. Sustain. Prod. Consum..

[bib69] Khan M.A., Mittal S., West S., Wuest T. (2018). Review on upgradability – a product lifetime extension strategy in the context of product service systems. J. Clean. Prod..

[bib70] Kharul R.v., Pomaje S.D. (1999).

[bib71] Kirchherr J., Reike D., Hekkert M. (2017). Conceptualizing the circular economy: an analysis of 114 definitions. Resour. Conserv. Recycl..

[bib72] Kirchherr J., van Santen R. (2019).

[bib73] Koenigsberg O., Kohli R., Montoya R. (2011). The design of durable goods. Market. Sci..

[bib74] Kumar V., Sezersan I., Garza-Reyes J.A., Gonzalez E.D.R.S., AL-Shboul M.A. (2019). Circular economy in the manufacturing sector: benefits, opportunities and barriers. Manag. Decis..

[bib75] Kunamaneni S., Jassi S., Hoang D. (2019). Promoting reuse behaviour: challenges and strategies for repeat purchase, low-involvement products. Sustain. Prod. Consum..

[bib76] Kwak M. (2016). Assessing the greenness of product lifetime extension. ICIC Express Letters, Part B: Applications.

[bib77] Kwak M., Kim H.M. (2012). Proceedings of the ASME 2012 International Design Engineering Technical Conferences & Computers and Information in Engineering Conference.

[bib78] Lacey E. (2009). Contemporary ceramic design for meaningful interaction and emotional durability: a case study. Int. J. Des..

[bib79] Landgraf R.W. (1987).

[bib80] Landgraf R.W., Conle A. (1984).

[bib81] Laurenti R., Sinha R., Singh J., Frostell B. (2015). Some pervasive challenges to sustainability by design of electronic products - a conceptual discussion. J. Clean. Prod..

[bib82] Li J., Shrivastava P., Zhang H.C. (2004). A distributed design methodology for extensible product life cycle strategy. IEEE Int. Symp. Electron. Environ..

[bib83] Liberati A., Altman D.G., Tetzlaff J., Mulrow C., Gøtzsche P.C., Ioannidis J.P.A., Clarke M., Devereaux P.J., Kleijnen J., Moher D. (2009). The PRISMA statement for reporting systematic reviews and meta-analyses of studies that evaluate healthcare interventions: explanation and elaboration. Br. Med. J..

[bib84] Lilley D., Bridgens B., Davies A., Holstov A. (2019). Ageing (dis)gracefully: enabling designers to understand material change. J. Clean. Prod..

[bib85] Lindgreen E.R., Salomone R., Reyes T. (2020). A critical review of academic approaches, methods and tools to assess circular economy at the micro level. Sustainability.

[bib86] Lobos A., Babbitt C. (2013). Integrating emotional attachment and sustainability in electronic product design. Challenges.

[bib87] Maitre-Ekern E. (2021). Re-thinking producer responsibility for a sustainable circular economy from extended producer responsibility to pre-market producer responsibility. J. Clean. Prod..

[bib88] Maitre-Ekern E., Dalhammar C. (2016). Regulating planned obsolescence: a review of legal approaches to increase product durability and reparability in Europe. Review of European, Comparative and International Environmental Law.

[bib89] Maldini I., Stappers P.J., Gimeno-Martinez J.C., Daanen H.A.M. (2019). Assessing the impact of design strategies on clothing lifetimes, usage and volumes: the case of product personalisation. J. Clean. Prod..

[bib90] Mesa J.A., Esparragoza I., Maury H. (2019). Modular architecture principles-MAPs: a key factor in the development of sustainable open architecture products. Int. J. Sustain. Eng..

[bib91] Mesa J., González-quiroga A., Maury H. (2020). Developing an indicator for material selection based on durability and environmental footprint : a Circular Economy perspective. Resour. Conserv. Recycl..

[bib92] Milios L. (2021). Overarching policy framework for product life extension in a circular economy—a bottom-up business perspective. Environmental Policy and Governance.

[bib93] Mohammadian S.H., Ait-Kadi D. (2010). Design stage confirmation of lifetime improvement for newly modified products through accelerated life testing. Reliab. Eng. Syst. Saf..

[bib94] Moher D., Liberati A., Tetzlaff J., Altman D.G. (2009). Preferred reporting items for systematic reviews and meta-analyses: the PRISMA statement. BMJ.

[bib95] Mongeon P., Paul-Hus A. (2016). The journal coverage of Web of Science and Scopus: a comparative analysis. Scientometrics.

[bib96] Mont O. (2008). Innovative approaches to optimising design and use of durable consumer goods. Int. J. Prod. Dev..

[bib97] Mont O., Dalhammar C., Jacobsson N. (2006). A new business model for baby prams based on leasing and product remanufacturing. J. Clean. Prod..

[bib98] Mugge R., de Jong W., Person O., Hultink E.J. (2018). ‘If it ain’t broke, don’t explain it’: the influence of visual and verbal information about prior use on consumers’ evaluations of refurbished electronics. Des. J..

[bib99] Mugge R., Schifferstein H.N.J., Schoormans J.P.L. (2010). Product attachment and satisfaction: understanding consumers’ post-purchase behavior. J. Consum. Market..

[bib100] Munson K., Yenal U., Lavelle P. (2018). Durability test design: linking fatigue and reliability. SAE Technical Papers.

[bib101] Munten P., Vanhamme J., Swaen V. (2021). Reducing obsolescence practices from a product-oriented PSS perspective : a research agenda. Reserche et Applications En Marketing.

[bib102] Nazlı T. (2021). Repair motivation and barriers model: investigating user perspectives related to product repair towards a circular economy. J. Clean. Prod..

[bib103] Nazzal D., Batarseh O., Patzner J., Martin D.R. (2013). Product servicing for lifespan extension and sustainable consumption: an optimization approach. Int. J. Prod. Econ..

[bib104] Nolan S., Woods R.A. (1989). Durability assessment in product development. SAE Technical Papers.

[bib105] Oguchi M., Tasaki T., Daigo I., Cooper T., Cole C., Gnanapragasam A. (2016).

[bib106] Page T. (2014). Product attachment and replacement: implications for sustainable design. Int. J. Sustain. Des.

[bib107] Pope S.M., Elliott J.R., Turbini L.J. (1998). Designing for technological obsolescence and discontinuous change: an evaluation of three successional electronic products. IEEE Int. Symp. Electron. Environ..

[bib108] Pretner G., Darnall N., Testa F., Iraldo F. (2021). Are consumers willing to pay for circular products ? The role of recycled and second-hand attributes , messaging , and third-party certification. Resour. Conserv. Recycl..

[bib109] Proske M., Finkbeiner M. (2019). Obsolescence in LCA–methodological challenges and solution approaches. Int. J. Life Cycle Assess..

[bib110] Proske M., Winzer J., Marwede M., Nissen N.F., Lang K. (2016).

[bib111] Raheja D. (2013). Heuristics for design for reliability for electrical and electronic products. IEEE Access.

[bib112] Rexfelt O. (2021).

[bib113] Richter J.L., van Buskirk R., Dalhammar C., Bennich P. (2019). Optimal durability in least life cycle cost methods: the case of LED lamps. Energy Efficiency.

[bib114] Rivera J.L., Lallmahomed A. (2016). Environmental implications of planned obsolescence and product lifetime: a literature review. Int. J. Sustain. Eng..

[bib115] Rizos V., Bryhn J. (2022). Implementation of circular economy approaches in the electrical and electronic equipment (EEE) sector: barriers, enablers and policy insights. J. Clean. Prod..

[bib116] Romero-Luis J., Carbonell-Alcocer A., Gertrudix M., Gertrudis Casado M. del C. (2021). What is the maturity level of circular economy and bioenergy research addressed from education and communication? A systematic literature review and epistemological perspectives. J. Clean. Prod..

[bib117] Sabbaghi M., Behdad S. (2017). Environmental evaluation of product design alternatives: the role of consumer’s repair behavior and deterioration of critical components. Journal of Mechanical Design, Transactions of the ASME.

[bib118] Sandborn P., Prabhakar V., Eriksson B. (2008). Proceedings of the ASME 2008 International Design Engineering Technical Conferences & Computers and Information in Engineering Conference.

[bib119] Satyro W.C., Sacomano J.B., Contador J.C., Telles R. (2018). Planned obsolescence or planned resource depletion? A sustainable approach. J. Clean. Prod..

[bib120] Sauerwein M., Doubrovski E., Balkenende R., Bakker C. (2019). Exploring the potential of additive manufacturing for product design in a circular economy. J. Clean. Prod..

[bib121] Schlegel M., Koch C., Mirtsch M., Harrer A. (2021).

[bib123] Singh J., Cooper T., Cole C., Gnanapragasam A., Shapley M. (2019). Evaluating approaches to resource management in consumer product sectors - an overview of global practices. J. Clean. Prod..

[bib124] Solomon R., Sandborn P., Pecht M. (2000). Electronic Part Life cycle concepts and obsolescence forecasting. IEEE Trans. Compon. Packag. Technol..

[bib125] Stamminger R., Bues A., Alfieri F., Cordella M. (2020). Durability of washing machines under real life conditions: definition and application of a testing procedure. J. Clean. Prod..

[bib126] Stamminger R., Tecchio P., Ardente F., Mathieux F., Niestrath P. (2018). Towards a durability test for washing-machines. Resour. Conserv. Recycl..

[bib127] Steeneck D.W., Sarin S.C. (2018). Product design for leased products under remanufacturing. Int. J. Prod. Econ..

[bib128] Su H. (2010). Automotive structural durability design using dynamic simulation and fatigue damage sensitivity techniques. SAE Technical Papers.

[bib129] Svensson-Hoglund S., Richter J.L., Maitre-Ekern E., Russell J.D., Pihlajarinne T., Dalhammar C. (2021). Barriers, enablers and market governance: a review of the policy landscape for repair of consumer electronics in the EU and the U.S. J. Clean. Prod..

[bib130] Tena D. (2021).

[bib131] Umeda Y., Daimon T., Kondoh S. (2007). Proceedings of ICED 2007, the 16th International Conference on Engineering Design, DS 42(August).

[bib132] Umemori Y., Kondoh S., Umeda Y., Shimomura Y., Yoshioka M. (2002).

[bib133] van den Bogaard J.A., Shangguan D., Jayaram J.S.R., Hulsken G., Brombacher A.C., Ion R.A. (2004). Using dynamic reliability models to extend the economic life of strongly innovative products. IEEE Int. Symp. Electron. Environ..

[bib134] van Desmet P., Krieken B., Aliakseyeu D., Mason J. (2012). 8th International Conference on Design and Emotion: Out of Control - Proceedings.

[bib135] van Nes N., Cramer J. (2005). Influencing product lifetime through product design. Bus. Strat. Environ..

[bib136] Vermunt D.A., Negro S.O., Verweij P.A., Kuppens D.v., Hekkert M.P. (2019). Exploring barriers to implementing different circular business models. J. Clean. Prod..

[bib137] Waage S.A. (2007). Re-considering product design: a practical “road-map” for integration of sustainability issues. J. Clean. Prod..

[bib138] Wallner T.S., Magnier L., Mugge R. (2020). An exploration of the value of timeless design styles for the consumer acceptance of refurbished products. Sustainability.

[bib139] Wallner T.S., Magnier L., Mugge R. (2022). Do consumers mind contamination by previous users ? A choice-based conjoint analysis to explore strategies that improve consumers’ choice for refurbished products. Resour. Conserv. Recycl..

[bib140] Wang C.J. (1990). Concept of durability index in product assurance planning. Proc. Annu. Reliab. Maintainab. Symp..

[bib141] Wang J.X., Burke H., Zhang A. (2022). Overcoming barriers to circular product design. Int. J. Prod. Econ..

[bib142] Wang W., Wang Y., Mo D., Tseng M. (2017). Component reuse in remanufacturing across multiple product generations. Procedia CIRP.

[bib143] Wei Z., Lin S., Luo L., Yang F., Konson D. (2012). Accelerated durability testing and data analysis for products with multiple failure mechanisms. Int. J. Reliab. Qual. Saf. Eng..

[bib144] Wei Z., Lin S., Luo L., Yang F., Konson D., Gurusamy B. (2013). Durability and reliability test planning and test data analysis. SAE International Journal of Materials and Manufacturing.

[bib145] Woolley M. (2003). Proceedings of the International Conference on Designing Pleasurable Products and Interfaces.

[bib146] Wrålsen B., Prieto-Sandoval V., Mejia-Villa A., O’Born R., Hellström M., Faessler B. (2021). Circular business models for lithium-ion batteries - stakeholders, barriers, and drivers. J. Clean. Prod..

[bib147] Xing K., Belusko M. (2008). Design for upgradability algorithm : configuring durable. Journal of Mechanical Design, Transactions of the ASME.

[bib148] Xu K., Xiong Y. (2008). 9th International Conference on Computer-Aided Industrial Design and Conceptual Design: Multicultural Creation and Design - CAIDCD 2008.

[bib149] Youn B.D., Choi K.K., Tang J. (2005). Structural durability design optimisation and its reliability assessment. Int. J. Prod. Dev..

[bib150] Yunus A.Z., Suleyman Z.M., Museib A.S. (2020). Improving the optimization of the life cycle of engineering products. International Journal of Advanced Science and Technology.

[bib151] Zallio M., Berry D. (2017). Design and planned obsolescence. Theories and approaches for designing enabling technologies. Des. J..

[bib152] Zeeuw Van Der Laan A., Aurisicchio M. (2019). Designing product-service systems to close resource loops: circular design guidelines. Procedia CIRP.

[bib153] Zhang B., Kimura F. (2005). Proceedings - Fourth International Symposium on Environmentally Conscious Design and Inverse Manufacturing, Eco Design 2005.

[bib154] Zhou J., Goel P.S. (1996). A framework for reliable and durable product design. SAE Technical Papers.

[bib155] Zhou W., Zheng Y., Huang W. (2017). Competitive advantage of qualified WEEE recyclers through EPR legislation. Eur. J. Oper. Res..

[bib156] Zink T., Geyer R. (2017). Circular economy rebound. J. Ind. Ecol..

